# Limited social plasticity in the socially polymorphic sweat bee *Lasioglossum calceatum*

**DOI:** 10.1007/s00265-018-2475-9

**Published:** 2018-03-10

**Authors:** P. J. Davison, J. Field

**Affiliations:** 1 School of Life Sciences, University of Sussex, John Maynard Smith Building, Brighton, BN1 9QG UK; 2 Present Address: Centre for Ecology and Conservation, University of Exeter, Penryn Campus, Cornwall, TR10 9EZ UK

**Keywords:** Sweat bee, *Lasioglossum*, Social phenotype, Field transplant, Social polymorphism

## Abstract

**Abstract:**

Eusociality is characterised by a reproductive division of labour, where some individuals forgo direct reproduction to instead help raise kin. Socially polymorphic sweat bees are ideal models for addressing the mechanisms underlying the transition from solitary living to eusociality, because different individuals in the same species can express either eusocial or solitary behaviour. A key question is whether alternative social phenotypes represent environmentally induced plasticity or predominantly genetic differentiation between populations. In this paper, we focus on the sweat bee *Lasioglossum calceatum*, in which northern or high-altitude populations are solitary, whereas more southern or low-altitude populations are typically eusocial. To test whether social phenotype responds to local environmental cues, we transplanted adult females from a solitary, northern population, to a southern site where native bees are typically eusocial. Nearly all native nests were eusocial, with foundresses producing small first brood (B1) females that became workers. In contrast, nine out of ten nests initiated by transplanted bees were solitary, producing female offspring that were the same size as the foundress and entered directly into hibernation. Only one of these ten nests became eusocial. Social phenotype was unlikely to be related to temperature experienced by nest foundresses when provisioning B1 offspring, or by B1 emergence time, both previously implicated in social plasticity seen in two other socially polymorphic sweat bees. Our results suggest that social polymorphism in *L. calceatum* predominantly reflects genetic differentiation between populations, and that plasticity is in the process of being lost by bees in northern populations.

**Significance statement:**

Phenotypic plasticity is thought to play a key role in the early stages of the transition from solitary to eusocial behaviour, but may then be lost if environmental conditions become less variable. Socially polymorphic sweat bees exhibit either solitary or eusocial behaviour in different geographic populations, depending on the length of the nesting season. We tested for plasticity in the socially polymorphic sweat bee *Lasioglossum calceatum* by transplanting nest foundresses from a northern, non-eusocial population to a southern, eusocial population. Plasticity would be detected if transplanted bees exhibited eusocial behaviour. We found that while native bees were eusocial, 90% of transplanted bees and their offspring did not exhibit traits associated with eusociality. Environmental variables such as time of offspring emergence or temperatures experienced by foundresses during provisioning could not explain these differences. Our results suggest that the ability of transplanted bees to express eusociality is being lost, and that social polymorphism predominantly reflects genetic differences between populations.

**Electronic supplementary material:**

The online version of this article (10.1007/s00265-018-2475-9) contains supplementary material, which is available to authorized users.

## Introduction

There is increasing interest in the environmental and genetic mechanisms underlying the transition from solitary living to eusociality (e.g. Yanega 1997; Field et al. 2010, 2012; Kapheim et al. 2012, 2015a; Kocher et al. 2013; Rehan and Toth 2015), and investigating these mechanisms requires taxa that straddle this transition (Field et al. 2010; Rehan and Toth 2015). Socially polymorphic sweat bees (Hymenoptera: Halictidae) are ideal models for this purpose, because different populations of the same species exhibit either eusocial or solitary behaviour (Soucy and Danforth 2002; Chapuisat 2010). In spring, mated females (foundresses) emerge from hibernation and excavate individual nest burrows. Each foundress then mass provisions a first brood (B1) of offspring in separate, sealed brood cells. In solitary populations, B1 offspring emerge to mate and females enter hibernation, becoming the following year’s new foundresses. In eusocial populations, however, at least some B1 females become workers that instead help to rear a second brood (B2) of reproductive offspring (Schwarz et al. 2007). Season length is thought to be a key proximate constraint on social phenotype because eusociality can be expressed only where the season is long enough to rear two consecutive broods (Soucy and Danforth 2002; Hirata and Higashi 2008; Field et al. 2010; Davison and Field, in preperation)

Within sweat bees, there have been at least two origins of eusociality and many subsequent losses, including to social polymorphism (Danforth 2002; Brady et al. 2006; Gibbs et al. 2012). It is thought that such reversals could be driven by selection acting on only a small number of regulatory switches (West-Eberhard 2003), and that in transitional populations initial plasticity in social phenotype might be lost once environmental conditions become predictable (Field et al. 2010; Cini et al. 2015; Smith et al. 2015). Therefore, a key question is to what extent alternative eusocial and solitary phenotypes result from environmentally mediated plasticity or represent distinct, genetically fixed alternatives (Wcislo 1997).

Field transplants are critical to addressing this question and yet are rarely performed (Yanega 1997; Field et al. 2012). Reciprocal transplants of the socially polymorphic sweat bee *Halictus rubicundus* Christ between social and solitary populations in the UK revealed that social phenotype is plastic with respect to the environment (Field et al. 2010, 2012). Evidence suggests that B1 females become workers only when emerging sufficiently early in the season (Field et al. 2010; see also Hirata and Higashi 2008), and that nest foundresses may be able to adjust the size of B1 offspring depending on anticipated social phenotype (Field et al. 2012, but see Field et al. 2010). Conversely, significant mitochondrial differentiation exists between eusocial and solitary populations of North American *H. rubicundus*, suggesting that social phenotype might have a fixed genetic component (Soucy and Danforth 2002). A laboratory common garden experiment also suggested that social phenotype may have a fixed genetic component in *Lasioglossum albipes* Fabricius (Plateaux-Quénu et al. 2000). However, fixed genetic differences between social phenotypes have never been demonstrated experimentally in a natural field setting, which is critical to fully account for unmeasured environmental variables (Yanega 1997; Field et al. 2012).

*Lasioglossum calceatum* Scopoli is a common and widespread socially polymorphic sweat bee of the Palearctic, closely related to *L. albipes* (Sakagami and Munakata 1972; Pesenko et al. 2000; Danforth et al. 2003; Davison and Field 2016). In this paper, we test for plasticity in social phenotype by transplanting foundresses from a northern, solitary UK population to a southern UK population where native bees are typically eusocial (see Davison and Field 2016 for site details). We also genotype offspring, which confirms previous work suggesting that *L. calceatum* expresses eusociality in the south of the UK (Davison and Field 2016).

We focus on three aspects of B1 female social phenotype: emergence time, pollen collection and body size. B1 offspring might become workers only if they emerge sufficiently early in the season (Hirata and Higashi 2008; Field et al. 2010), workers typically begin provisioning the natal nest within 1 or 2 days of emergence **(**PJD, personal observation), and in common with most other eusocial sweat bees, B1 workers tend to be smaller than their mothers (Packer and Knerer 1985; Davison and Field 2016). Critically, since offspring are mass provisioned, B1 body size may largely reflect investment decisions by foundresses at the time of provisioning (Plateaux-Quénu 1983; Richards and Packer 1994). If social phenotype is plastic, foundresses transplanted to the south from northern solitary populations might respond by producing small B1 offspring that in turn remain at their nest as workers, or perhaps initiate their own nests instead of entering hibernation (Field et al. 2010, 2012). Instead, we find that most transplanted foundresses and their offspring show no evidence of plasticity, indicating that inter-population differences in social phenotype predominantly reflect genetic differentiation.

## Methods

### Transplants

**Fig. 1:**
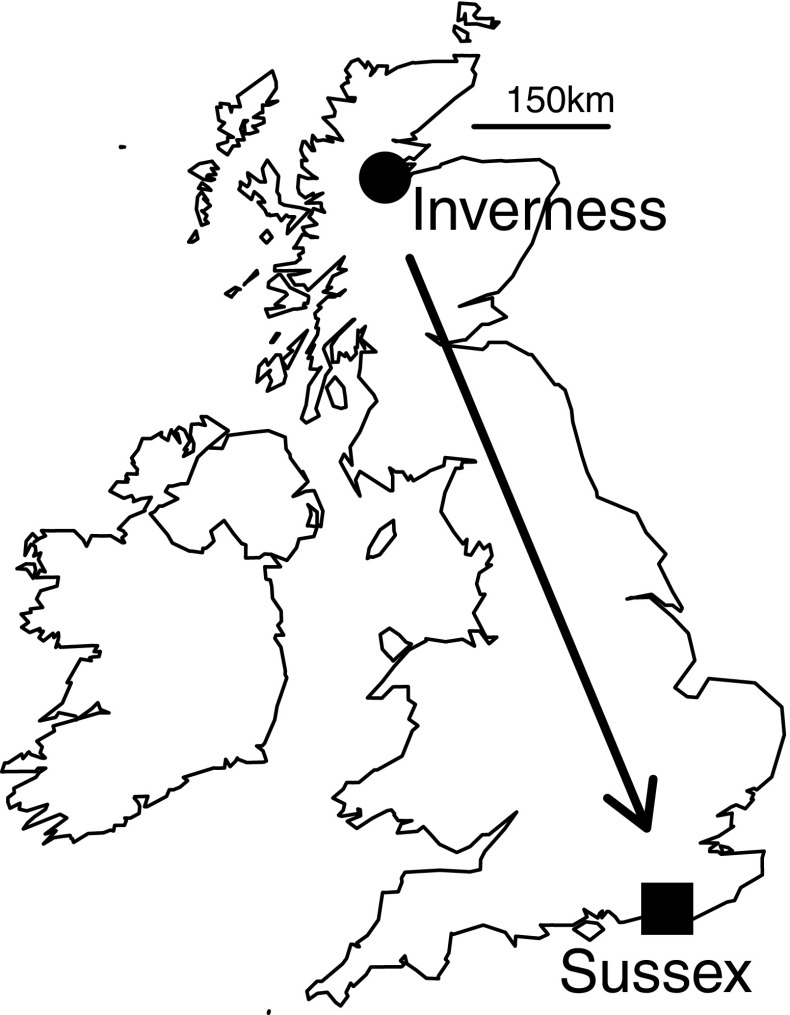
Map of the UK and Ireland showing the location of our two study sites. Adult females were collected at Inverness and transplanted to Sussex

Foundresses were transplanted from Inverness, in the far north of the UK, approximately 800 km south to a nesting aggregation at the University of Sussex campus (Sussex) where annual temperatures are higher and the season is longer (Fig. 1; Table 1). Bees at Inverness nest solitarily but bees at Sussex typically express eusociality (Davison and Field 2016). Because nesting aggregations are hard to find (Richards et al. 2015), we were unable to perform a control transplant from another eusocial site in the present study. However, controls implemented in previous studies show that transplantation per se is unlikely to influence behaviour (Field et al. 2010, 2012; Davison and Field, in preparation). Bees were transplanted from Inverness on 15–16 August 2014 (autumn transplant) and 16 May 2015 (spring transplant). Autumn-transplanted bees (*n* = 70) were freshly emerged B1 females caught returning to their nests from feeding/mating flights. Spring-transplanted bees (*n* = 202) were nest foundresses recently emerged from hibernation, and were caught returning from feeding or provisioning flights. Most spring transplants were not carrying pollen and were therefore unlikely to have already begun provisioning their own offspring.

**Table 1:** Details of locations used in the study, showing key environmental variables thought to influence social phenotype

Location	Latitude/longitude	Temperature^a^ (°C)	Season length^b^ (months)	Altitude (masl)	Native social phenotype
Sussex	50.864/− 0.084	17.4	6.1	82	Eusocial
Inverness	57.554/− 4.456	13.4	4.8	5	Solitary

^a^Mean annual land surface temperature 1981–2006 (data from Hay et al. 2006)

^b^Estimate of the time available for nesting during the active season, calculated from the mean number of days during the year between 1981 and 2006 on which the land surface temperature exceeds 16 °C (see Davison and Field 2017 for methodology)

Bees were caught with an insect net and marked on both the clypeus and thorax with a single spot of enamel paint (Revell® and Humbrol™ enamel model paints), denoting the time of transplant (autumn or spring). Thus, transplanted bees could be readily distinguished from native Sussex bees. Prior to release, bees were maintained inside individual plastic tubes either in a cold box containing ice packs or a fridge at 4 °C. Autumn-transplanted bees were released directly into 14 L plastic buckets containing artificial nest holes (15–20 cm), which were embedded into the ground near to the nesting aggregation at Sussex. Each bucket was covered with netting to encourage bees to enter the holes. Netting was removed the following morning and bees allowed to fly freely inside an insect-proof cage containing flowers before entering hibernation inside the buckets, which were left embedded in the ground throughout the winter. Buckets were removed and re-embedded within the Sussex nest aggregation before the start of spring 2015. Spring-transplanted bees were released over three evenings (17–19 May 2015) directly into artificial nest holes created among native and autumn-transplanted nests within the Sussex aggregation (not in the buckets). Of the two autumn-transplanted bees that successfully founded nests (see “Results”), one nested within a bucket and one in the ground surrounding the buckets. Similarly, some spring-transplanted foundresses also founded nests in buckets, while others utilised the artificial nest burrows or dug new nest burrows in the surrounding soil.

### Foundress demography and body size

We recorded the timing of three key events for native and transplanted bees: (1) date of nest initiation (the first day on which a foundress provisioned), (2) date of first B1 female emergence and (3) the time taken to produce the first B1 female offspring (time between (1) and (2)).

Foundresses initiating nests in spring were caught with an insect net after being observed provisioning, and individually marked with unique colour combinations of two enamel paint spots (Revell® and Humbrol™) applied to the thorax with a pin. Wing length was measured to the nearest 0.1 mm with digital callipers, as the distance between the outer edge of the tegula and the end of the forewing. Nest entrances were marked using individually numbered nails. During the foundress provisioning phase, the nesting aggregation was divided into two sections, which were observed continuously by the same person on alternate days when the weather was suitable for foraging (*n* = 29 observation days). It was not possible to record data blind because our study involved focal animals in the field.

The timing of nest initiation and offspring emergence date can vary between years, and therefore as a comparison we utilise demographic data from two previous years in which native *L. calceatum* were studied at Sussex: 2012 and 2013. Data in these years were collected using the same methods as in the present study (see Davison and Field 2016 for details).

### Determining social phenotype and offspring size

Nests were considered eusocial only if B1 females were observed provisioning. Workers were identified as unmarked bees provisioning a nest in which the foundress had been marked: workers begin provisioning within 1 or 2 days of emergence, whereas B1 females that directly enter hibernation are typically observed entering the nest for several days after emergence but never with pollen (Davison and Field 2016). B1 females were caught on emergence from their nest only after being observed provisioning. They were then measured and marked with a single paint spot on the clypeus and thorax, using a colour unique within their nest. Directly hibernating offspring were measured when excavated from beneath their nests at the end of the season (see below). With the help of a second observer, nests in both sections were continually observed during the worker-provisioning phase (*n* = 26 observation days).

### Nest excavations

**Fig. 2:**
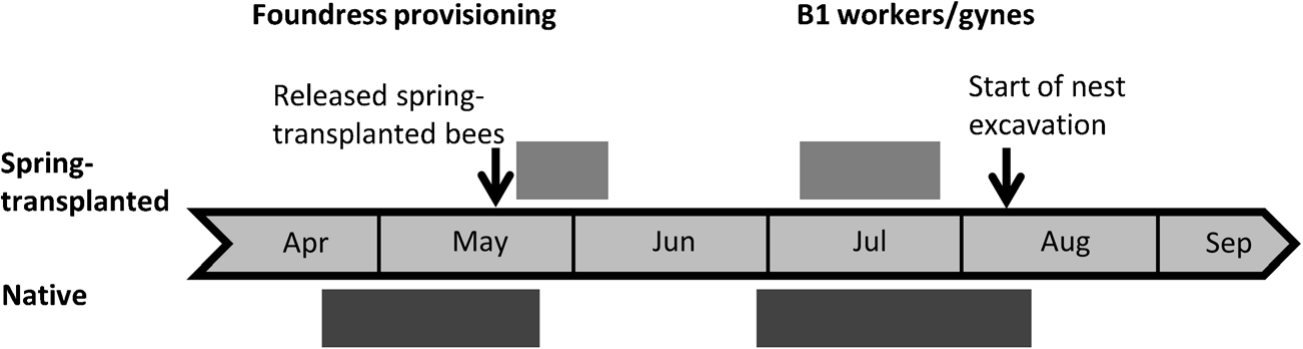
The timing and duration of key events for spring-transplanted Inverness (light grey) and native Sussex (dark grey) nest foundresses and their offspring. Solid bars show the periods during which activity was observed, and represent all bees in that cohort. Not all bees within each cohort, represented by a bar, began or finished individual stages on the same day. Bars therefore represent the first and last days on which different individual bees within a cohort were observed. Gaps between bars shows periods of bee inactivity

Nests were excavated from 6 to 15 August 2015, near to the end of the season but prior to the emergence of B2 offspring (Fig. 2). All brood and adult bees (B1 females and foundresses) were removed and stored in ethanol before genotyping. In nests of *L. calceatum*, cells forming each brood are arranged in a single cluster surrounded by a cavity (Sakagami and Michener 1962), and it was therefore possible to be certain that all brood in a given nest had been collected. Excavations were continued well below the level of brood cell clusters to detect hibernating B1 offspring.

### DNA extraction and microsatellite analysis

DNA was extracted from whole bees using the ammonium acetate precipitation method (Nicholls et al. 2000). We amplified 12 microsatellite loci, originally developed for *L. malachurum*, in two multiplexes (Parsons et al. 2017; Table S1). Multiplexes were amplified in a 2 μL Qiagen Multiplex reaction using the following PCR profile: 95 °C for 15 min, followed by 44 × (94 °C for 30 s, 57 °C for 90 s and 72 °C for 60 s), then 60° for 30 min. PCR products were genotyped using an ABI 3730 48-well capillary DNA Analyser using LIZ size standard (Applied Biosystems Inc.), and alleles were scored using Genemapper® v3.7 software. Two microsatellites were monomorphic and were discarded. The remaining ten loci had 6–19 alleles per locus ($\overline{x}$ = 9.5 alleles per locus) across both populations (see Table S2 for breakdown by population). We tested for linkage disequilibrium (LD) and departure from Hardy-Weinberg equilibrium (HW) within and among the Sussex and Inverness populations using Genepop 4.2 (Raymond and Rousset 1995; Rousset 2008). For these tests, we selected one female from each nest at Sussex to avoid pseudoreplication. To correct *p* values in multiple tests, we applied the *q* value to LD *p* values using QVALUE (Storey 2002). The *q* value is a measure of significance in terms of false discovery rate unlike the conventional Bonferroni correction, which attempts to measure significance in terms of false positives only (Storey 2002), and therefore provides a more powerful method for correcting multiple tests (Verhoeven et al. 2005). As a measure of genetic diversity, we recorded the total number of alleles at a given locus, and the observed and expected heterozygosity.

### Brood relatedness

We used the software Relatedness 5.0.8 (Queller and Goodnight 1989) to estimate the life-for-life coefficient of relatedness (*r*) among B2 females and between foundresses and B2 females within each nest. Allele frequencies were estimated and calculations performed when weighting nests equally. Standard errors and 95% confidence intervals were obtained by jackknifing over nests (Queller and Goodnight 1989). We separated female B2 brood into groups of full sisters using the computer program Kinship 1.3.1 (Goodnight and Queller 1999). We asked whether females were more likely to be full sisters (*r* = 0.75) than aunt-niece (*r* = 0.375), with 100,000 replicates to estimate significance values: any B2 female brood laid by a B1 female will be the niece of a B2 female brood laid by the foundress. Where the principle egg-layer’s genotype was available, or could be reconstructed, we assigned male production: males were allocated to the principle egg-layer if they shared one of her two alleles at each locus, and to a secondary female if one or more alleles were not shared.

### Confirming offspring population of origin

*Lasioglossum* offspring typically hibernate beneath their natal nest (Sakagami and Fukuda 1972), and B1 offspring of transplanted foundresses were indeed frequently found hibernating beneath their natal nests. In nests where we were unable to match the genotypes of B1 offspring with the foundress, however, we used STRUCTURE (version 2.3.4; Pritchard et al. 2000) to confirm that these hibernating adults were not offspring of native foundresses that might have entered the nest. STRUCTURE divides genotypes into genetic clusters according to HW and LD. Using this method, we could also test whether two of three bees (the third was not genotyped) that initiated new nests during the worker-provisioning phase originated from native or transplanted nests. We assumed admixture and uncorrelated allele frequencies, and specified the number of possible genetic clusters as *K =* 1–3. We ran three replicates for each *K*, and specified a burn-in period of 100,000 steps. A single individual from each nest was included, together with additional adults caught at Inverness in spring 2015 but not released at Sussex (*n* = 21) and additional bees from the Sussex native population (*n* = 18). We implemented the Evanno method (Evanno et al. 2005) within the program Structure Harvester (Earl and Vonholdt 2012) to determine the best fitting value of *K*. We further characterised genetic differentiation between our Inverness and Sussex *L. calceatum* populations by calculating *F*_ST_ using the default settings in Genepop 4.2 (Raymond and Rousset 1995; Rousset 2008).

### Data analysis

To determine whether transplanted foundresses and their offspring exhibited plasticity, we examined two characteristics associated with social phenotype: worker behaviour and B1 offspring size.

First, we tested whether the observed pattern of behaviour exhibited by offspring of native and transplanted foundresses indicated (i) environmentally mediated plasticity or (ii) fixed genetic differences between the two populations. Under plasticity, the timing of B1 emergence might be a key factor mediating the decision of B1 offspring to become workers (Field et al. 2010). A significant effect of ‘source’ (Sussex or Inverness) on social phenotype would indicate fixed genetic differences between populations, whereas a significant effect of ‘emergence date’ would be indicative of plasticity. Spring-transplanted foundresses typically provisioned to produce their B1 offspring later than native foundresses (Fig. 2; Fig. 5a), and therefore could have experienced different environmental conditions that may have influenced offspring social phenotype. To control for one such factor, we included temperature during a foundresses’ provisioning period in the model. This was calculated as the average of mean daily temperature for each day between a foundress’s first and last observed provisioning events, yielding ‘foundress provisioning temperature’. We analysed the effect of ‘source’, ‘emergence date’ (designated as the date on which the first B1 female emerged at each nest) and ‘foundress provisioning temperature’ on a nest’s ‘phenotype’ (presence or absence of workers) using a generalised linear model (GLM) with binomial errors. Given that later-provisioned offspring also emerged later (Fig. 5a), we checked for collinearity among explanatory variables (Dormann et al. 2013) by examining variance inflation factor (VIF) scores using the function ‘vif’ in the R-Package ‘car’ (Fox and Weisberg 2011). We employed a conservative threshold of VIF ≥ 2.5 to identify collinearity (Allison 2012). For all variables, VIF scores were low (< 1.3), indicating no significant collinearity.

Second, because eusociality in *L. calceatum* is associated with B1 offspring (workers) that are significantly smaller than their mothers (caste-size dimorphism; Davison and Field 2016), we tested for caste-size dimorphism and examined whether ‘source’ affected the size of B1 female offspring produced by native and transplanted foundresses. As there were multiple offspring per nest, we used a generalised linear mixed model (GLMM) to test for effects of ‘caste’ and ‘source’ on ‘female wing length’, with ‘nest’ included as a random factor. We initially included a caste/source interaction to test whether foundresses from different sources produced offspring of different sizes relative to themselves.

We also tested for differences between native and transplanted bees in the time taken to produce the first B1 offspring. We used a GLM with normal errors to test for effects of ‘first foundress provision date’ and ‘source’ on ‘development time’. Development time was considerably left skewed, and so we implemented the function *powerTransform* in the R-package ‘car’ (Fox and Weisberg 2011) to transform the data. Offspring of transplanted and native foundresses were different in size, and therefore we included ‘size’ to control for this difference. As a measure of size, we used the mean wing length of marked workers in eusocial nests, and of B1 females excavated at the end of the season from solitary nests.

We used a GLM with negative binomial errors to examine the relationship between the number of workers in a nest and ‘productivity’ (the number of immature B2 offspring produced), with ‘number of workers’ as the single explanatory variable.

We used Chi-squared tests with Yates’ correction to compare the frequency of nest failure between nests initiated by native versus transplanted foundresses, and to compare the frequency of successful spring nest initiation between autumn and spring-transplanted Inverness bees. Foundresses were considered to have successfully initiated nests once they had started provisioning, and nest failure was indicated by the absence of detected B1 offspring.

For all models, we report significance values when removing terms from the minimal adequate model, after stepwise reduction from the maximal model (Crawley 2013). All analyses were conducted in the *R* environment (R Development Core Team 2013). Results are presented ± 1 standard error.

All data generated or analysed during this study are included in this published article and its supplementary information files.

## Results

### Nesting success of transplanted bees

Spring and autumn-transplanted foundresses were equally likely to initiate nests at Sussex (Pearson’s chi-squared test: *X*^2^ = 0.862, *p* = 0.353, *n* = 4/70 autumn-transplanted (5.7%), *n* = 21/202 spring-transplanted (10.4%)). Of these, approximately half successfully produced B1 offspring (*n* = 2/4 autumn-transplanted, *n* = 10/21 spring-transplanted). The rate of failure to produce at least one detected B1 offspring did not differ between nests initiated by native and transplanted foundresses (Pearson’s chi-squared test: *X*^2^ = 0.019, *p* = 0.891, native = 56.6%, *n* = 53 nests; transplanted = 52.4%, *n* = 25).

### Social phenotype

Social phenotype was successfully recorded at 39 nests (*n* = 29 native, *n* = 10 spring-transplanted). Nearly all native nests (*n* = 28/29) were social, with B1 female offspring that began provisioning as workers ($\overline{x}$ = 3.1 ± 0.33 B1 workers per nest). In contrast, nine out of ten nests initiated by spring-transplanted Inverness foundresses did not become social (Fig. 4a). The two successful nests initiated by autumn-transplanted foundresses also did not become social: one produced two B1 males and the other a single B1 female that brought one recorded pollen load to the nest before disappearing. Although social phenotype was somewhat ill defined at these nests, neither scenario was observed among the 29 native nests.

**Fig. 3:**
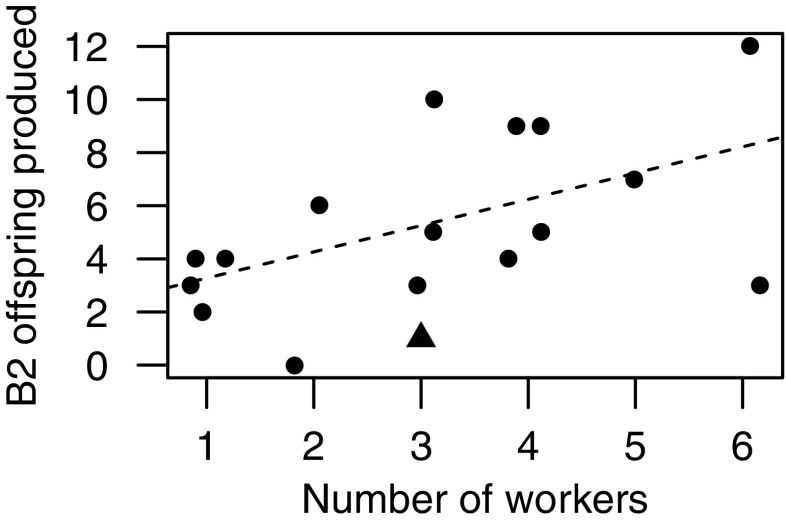
Relationship between the number of provisioning workers recorded a nest and the number of B2 male and female offspring produced. Filled circles represent native nests, and the filled triangle the single social transplanted nest. Points are jittered to reveal multiple overlapping data points. The dashed line shows least-squares regression for native nests

Foundresses were alive at the time of B1 female emergence in six of the nine solitary nests initiated by spring-transplanted foundresses, and these foundresses were regularly observed leaving the nest on nectar-collecting trips alongside B1 females. The single transplanted foundress whose nest became social produced three B1 females, all of which began provisioning in the presence of the foundress. Provisioning behaviour by B1 females was clear-cut at this nest because each B1 female was observed provisioning on at least ten occasions over three or more separate days (Yanega 1989). In contrast with the solitary nests, and in common with native eusocial nests, the foundress did not leave her nest after B1 offspring emergence. Among native nests, the number of B2 offspring produced increased linearly with the number of workers in a nest (GLM: *X*^2^_1,15_ = 4.944, *p* = 0.026; Fig. 3). The single eusocial nest initiated by a spring-transplanted foundress produced just two B2 offspring, despite having three B1 provisioners. Three native nests containing three workers produced three, five, and ten B2 offspring, respectively (Fig. 3).

### Bee size

**Fig. 4:**
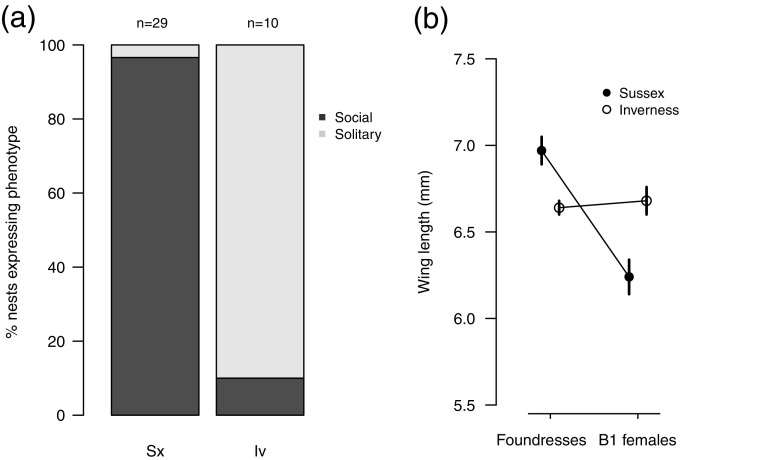
**a** Proportion of native nests at Sussex (Sx) and nests initiated by spring-transplanted foundresses from Inverness (Iv) that expressed social or solitary behaviour. **b** Mean wing lengths (mm) of native (Sussex) and transplanted (Inverness) foundresses and their B1 female offspring (± 1SE). Foundresses: *n* = 18 from Sussex, *n* = 5 from Inverness. B1 females: *n* = 51 from Sussex, *n* = 13 from Inverness. Significant caste/source interaction *X*_21_ = 20.302, *p* < 0.001

Native foundresses produced B1 females significantly smaller than themselves. In contrast, transplanted foundresses produced offspring the same size as themselves, and which were larger than native B1 females (GLMM: caste/source interaction *X*^2^_1_ = 20.302, *p* < 0.001; Fig. 4b). Two of the three B1 offspring produced in the single eusocial nest initiated by a transplanted foundress were the same size as native workers, while the third was closer in size to the mean for transplanted foundress’ B1 offspring. A B1 female offspring excavated from beneath the single native solitary nest was similar in size to other native B1 females that became workers. All of these offspring are included in the analysis illustrated in Fig. 4b.

### Foundress provisioning, offspring emergence date and development time

**Fig. 5:**
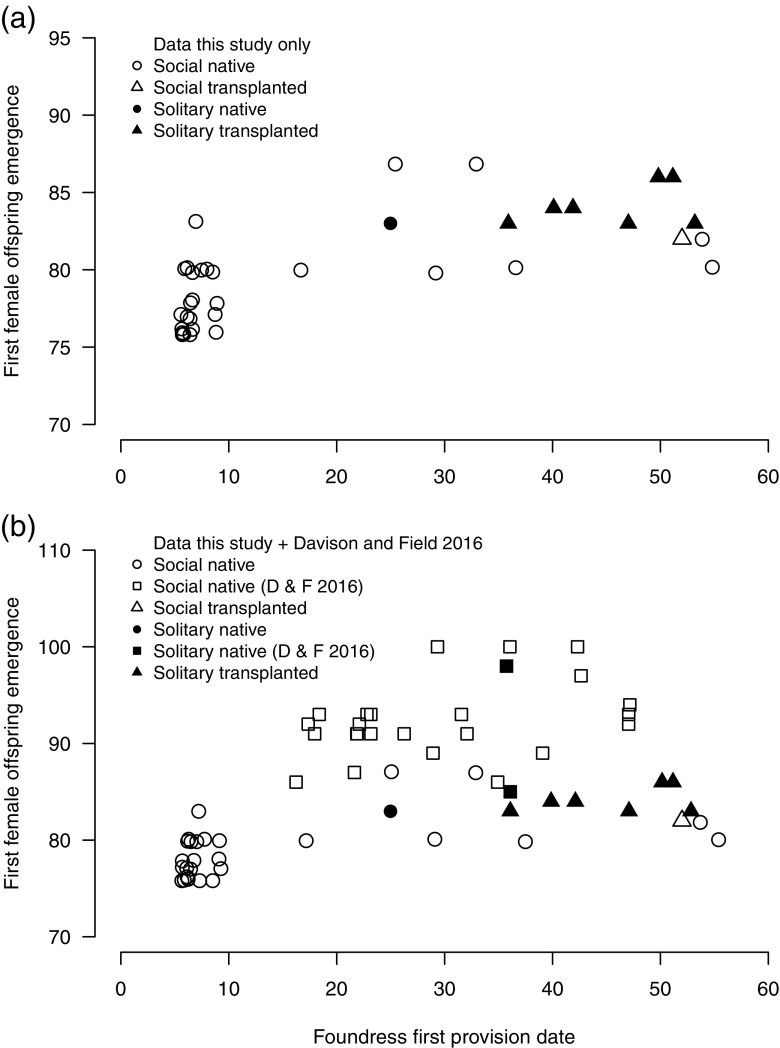
Social phenotype and the relationship between the date a foundress was first observed to provision in spring and the emergence date of her first female offspring. Panels show **a** data from the present study *only* and **b** data from the present study *together with* data gathered in the same way by Davison and Field (2016) for comparison, representing native bees studied in 2012 and 2013. Symbols denote social phenotype and source: open symbols show social nests where at least some offspring became workers, and filled symbols solitary nests where no female B1 offspring became workers. Triangles denote bees transplanted from Inverness, and circles and squares native bees from Sussex. See keys for details. Points are jittered to reveal multiple overlapping observations. Day zero is 14 April and day 70 is 23 June, standardised across years

Native Sussex foundresses were first observed provisioning on 20 April 2015, with an average first provisioning date of 24 April ± 1.4 days (*n* = 51 native foundresses). However, because the season started later in Inverness than at Sussex in 2015, and because spring transplants could not be carried out until foundresses emerged from hibernation in spring, the first spring-transplanted foundresses did not begin provisioning until 20 May (Fig. 2; Fig. 5a). Nevertheless, two native Sussex foundresses did begin provisioning *after* spring-transplanted foundresses (Fig. 5a) and had eusocial nests. Moreover, four spring-transplanted foundresses that did not produce workers still began provisioning before the latest-provisioning eusocial foundresses in 2012 and 2013 (Fig. 5b). The two autumn-transplanted foundresses that established nests began provisioning on 21 April and 9 May, respectively.

Time taken to produce the first female offspring did not differ between nests initiated by native and transplanted foundresses, and decreased linearly as the date when a foundress first started provisioning progressed (GLM: F_1,30_ = 292.58, *p* < 0.001). Nevertheless, because their nests were generally initiated later in spring, the first female offspring of transplanted foundresses emerged later than those from most native nests (Fig. 5a; Wilcoxon signed rank test: W = 27.5, *p* < 0.001, *n* = 10 transplanted, *n* = 31 native nests), although at an earlier date than almost all native offspring in two previous years (Fig. 5b). However, nests of transplanted bees may not have been solitary simply because B1 offspring emerged later in the season, or because transplanted foundresses provisioned later in the spring: four nests of native foundresses had been initiated later, or had B1 females that emerged later, yet still became eusocial (Fig. 5a). Indeed, even after controlling for the effects of temperature during foundress provisioning, and for offspring emergence date, nests initiated by native foundresses were still significantly more likely to become eusocial than nests initiated by transplanted foundresses (GLM: foundress provisioning temperature *X*^2^_1,34_ = 0.739, *p* = 0.390; B1 emergence date *X*^2^_1,34_ = 1.613, *p* = 0.204; bee source *X*^2^_1,34_ = 5.565, *p* = 0.021). Additionally, B1 female offspring of transplanted foundresses in the present study emerged relatively early compared with native eusocial B1 offspring from two previous years in the same nest aggregation at Sussex (Fig. 5b; Davison and Field 2016).

### Brood genotyping

Prior to *q* value correction, three locus pairs were weakly significant for LD. After *q* value correction, however, there was no significant LD within or between the Sussex and Inverness populations. One locus deviated from HW at both Sussex and Inverness (LMA53, see Table S2). Genetic diversity was variable among loci, with a mean expected heterozygosity of 0.66 at Sussex and 0.54 at Inverness (Table S2).

**Fig. 6:**
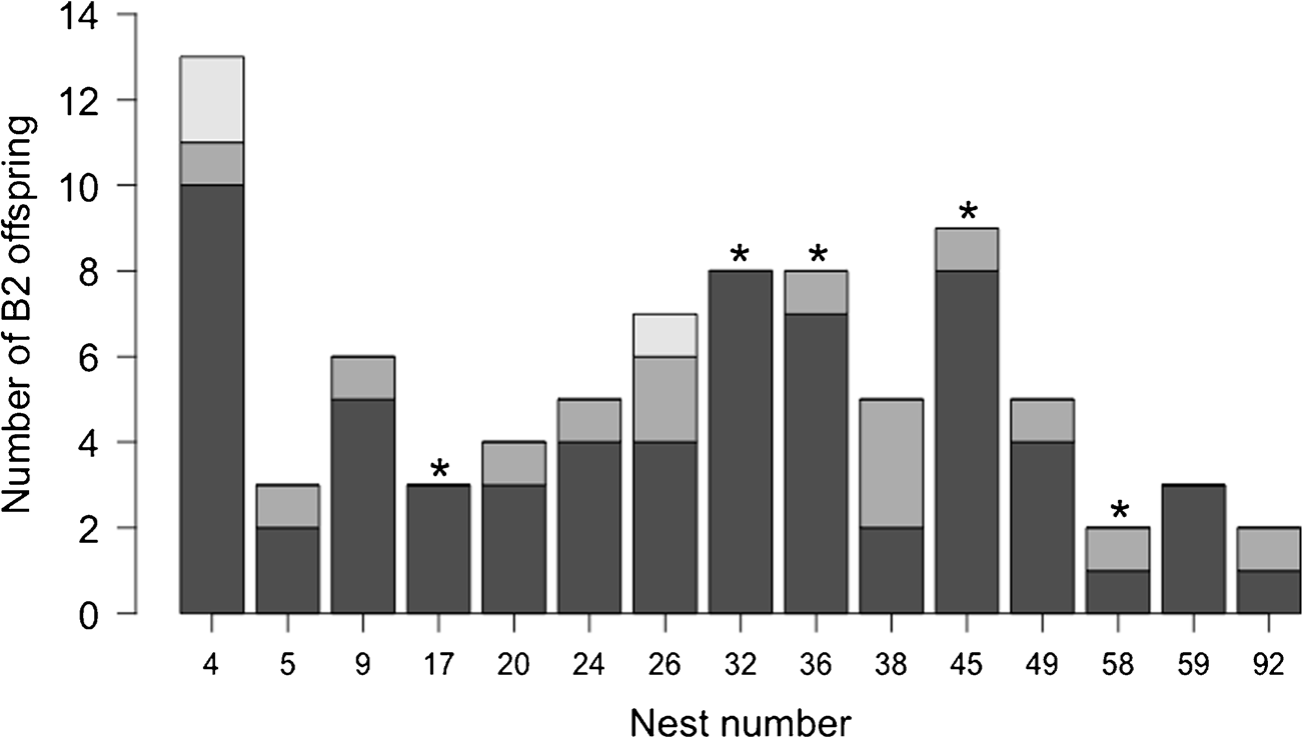
Partitioning of B2 offspring produced in social nests initiated by native foundresses at Sussex. Each nest is represented by a bar, and offspring from different mothers are represented by blocks within each bar. Asterisks indicate nests in which the foundress was alive at the time of excavation (see Table 2 and Table S3 for details)

We successfully genotyped B2 offspring from 22 nests (*n* = 15 native, *n* = 7 transplanted). Native nests contained a mean of 5.7 genotyped B2 offspring per nest ($\overline{x}$ = 3.7 ± 0.67 females and $\overline{x}$ = 1.9 ± 0.41 males). Five nests also contained a live foundress and five contained live workers at the time of excavation. Genetic data confirm our behavioural observations that *L. calceatum* exhibits eusociality at Sussex: average relatedness among B2 female brood within nests was 0.74 ± 0.03 (mean ± SE; 95% CI [0.67; 0.81]) (see Table 2 for a breakdown by nest). In four of five nests containing a live foundress at excavation, the foundress monopolised most or all B2 reproduction (Fig. 6; Table 2). Of native nests containing multiple B2 female brood (*n* = 13/15), approximately half (*n* = 6/13) contained a single B2 female which was not sister to the remaining female brood (Fig. 6). Six of 12 nests in which it was possible to assign males contained males not laid by the principal egg-layer (Fig. 6; Table S3). We found no evidence of multiple mating by native foundresses, suggesting that Sussex *L. calceatum* are monandrous. However, we found evidence of at least one alien bee reproducing within a single nest (see nest 58 in Table 2).

**Table 2:** Numbers of genotyped brood and average relatedness among B2 female offspring within nests initiated by native foundresses at Sussex. Relatedness between a foundress and B2 female offspring is included for nests where the foundress was found alive at the time of nest excavation

Nest	# female B2	# male B2	Total B2 genotyped	F present?	r among B2 females [95% CI]	r foundress to B2 females
4	11	2	13	0	0.88 [0.78; 0.99]	–
5	2	1	3	0	1 [1; 1]	–
9	0	6	6	0	–	–
17	3	0	3	1	0.84 [0.67; 1.01]	0.67 [0.39; 0.96]
20	4	0	4	0	0.69 [0.44; 0.94]	–
24	3	2	5	0	0.84 [0.65; 1.03]	–
26	5	2	7	0	0.72 [0.51; 0.93]	–
32	5	3	8	1	0.93 [0.82; 1.04]	0.31 [0.05; 0.56]
36	5	3	8	1	0.77 [0.58; 0.95]	0.42 [− 0.23; 1.07]
38	2	3	5	0	0.88 [0.71; 1.06]	–
45	8	1	9	1	0.75 [0.60; 0.90]	0.23 [− 0.39; 0.85]
49	4	1	5	0	0.29 [− 0.09; 0.67]	–
58	1	1	2	1	–	−0.19 [− 0.57; 0.20]
59	3	0	3	0	0.83 [0.67; 0.99]	–
92	2	0	2	0	0.25 [− 0.04; 0.54]	–

We successfully genotyped the foundress, a marked B1 female and two female B2 offspring from the single eusocial nest initiated by a spring-transplanted Inverness foundress. The foundress was the mother of the B1 female. However, the two female B2 offspring were not sisters, and our data suggest two alternative possibilities for parentage: (i) the foundress mated multiply and laid both, or (ii) a B1 female laid one. In either case, bees in this nest exhibited eusocial behaviour not previously recorded at Inverness (Davison and Field 2016). We also genotyped female B1 offspring from six solitary nests of transplanted foundresses ($\overline{x}$ = 1.8 per nest). In the nests where we genotyped the foundress, she matched as mother to most or all of the adult females excavated from her nest. The population of origin for B1 females in the remaining four nests was determined by the STRUCTURE analysis (see below).

The STRUCTURE analysis strongly supported the existence of the two known populations (*K* = 2, Sussex and Inverness), and assigned all bees of known origin to the correct cluster (Fig. S1). The pairwise *F*_ST_ value for our Inverness and Sussex was 0.286, indicating considerable genetic differentiation between our populations (Soro et al. 2010). All B1 offspring excavated from beneath the nests of transplanted foundresses were assigned to the cluster containing bees from Inverness. The two genotyped bees that initiated new nests in the summer were assigned to the Sussex population. Independent summer nest founding has not previously been reported (Davison and Field 2016), and therefore represents the discovery of a new behaviour by *L. calceatum* at Sussex.

## Discussion

Few studies have utilised field transplants to address the mechanisms underlying socially polymorphic behaviour (Field et al. 2010, 2012, see also Cronin 2001; Baglione et al. 2002). We transplanted the socially polymorphic sweat bee *Lasioglossum calceatum* from a non-eusocial population at Inverness, in the far north of the UK, 800 km south to a predominantly eusocial population on the University of Sussex campus, in the far south of the UK (Fig. 1). Most native Sussex bees exhibited eusociality, whereas nine of ten transplanted Inverness bees and their offspring exhibited solitary behaviour. Bearing in mind that the sample size is small, we do not wish to overemphasise these precise figures, but our best estimate is that 10% of Inverness bees are capable of expressing eusociality. Our results provide the first field-based experimental evidence that inter-population differences in social phenotype might predominantly reflect genetic differentiation, and provide genetic confirmation that *L. calceatum* is truly eusocial in the southern UK.

In nine of ten cases, neither spring-transplanted foundresses nor their offspring showed evidence of social plasticity: spring-transplanted foundresses provisioned large B1 female offspring that did not attempt to become workers. By contrast, native foundresses produced small B1 females that typically became workers (Fig. 4a, b). We confirmed that offspring of transplanted foundresses did not enter hibernation simply because their mothers had died (e.g. Packer 1990; Field et al. 2010), since transplanted foundresses were still alive in seven of ten nests at the time of offspring emergence.

One possibility, however, is that because spring-transplanted foundresses developed and overwintered at the Inverness source site, solitary behaviour represents plasticity in response to cues experienced by the foundress prior to transplantation (Thibert-Plante and Hendry 2011). Maternal effects may then influence offspring social phenotype, for example through nutrition provided by mothers (e.g. Brand and Chapuisat 2012; Kapheim et al. 2015b; Berens et al. 2015). However, relatively large B1 females can still become workers in other socially polymorphic sweat bees (Field et al. 2010), and small B1 females can enter hibernation if they emerge late in the season (Hirata and Higashi 2008). This suggests that in species exhibiting plasticity, any nutrition-mediated maternal effects can be overridden by environmental cues experienced by emerging offspring. Furthermore, although we could not be certain of social phenotype, two autumn-transplanted foundresses successfully founded nests that did not become social: one produced two B1 males, and the other a single female that provisioned once before disappearing. Neither scenario was observed among native nests, 28 out of 29 of which became eusocial. These foundresses experienced overwintering conditions at Sussex, yet neither nest became social as expected if social phenotype was plastic, which together with the spring-transplanted nest that became social hints that overwintering conditions alone are unlikely to explain our results. We note the possibility that the male-producing transplanted foundress had not mated prior to capture in the previous autumn.

Another possibility is that emerging later than most native B1 females may have increased the propensity for B1 females of transplanted foundresses to enter hibernation instead of becoming workers (Field et al. 2010; Fig. 5a). Nevertheless, they still emerged earlier in the season than almost all native B1 workers in two previous years (Fig. 5b; Davison and Field 2016), and our analysis showed that neither temperature experienced by foundresses during provisioning nor offspring emergence date successfully explained social phenotype. Moreover, although spring-transplanted foundresses tended to begin provisioning later than Sussex foundresses in this study, four spring-transplanted foundresses that did not produce workers still began provisioning before the latest-provisioning eusocial foundresses in 2012 and 2013 (Fig. 5b). Together this suggests that date of offspring emergence per se might not be a critical factor influencing the social phenotype of *L. calceatum*. However, we cannot discount the possibility that other unmeasured cues correlated with later foundress provisioning/offspring emergence could have influenced social phenotype.

### Plasticity and its loss

Our results show that although most were solitary, transplanted Inverness bees can still express eusociality (see also Plateaux-Quénu et al. 2000): all three B1 female offspring of one spring-transplanted foundress began provisioning the natal nest, behaviour never previously observed at Inverness (Davison and Field 2016). Moreover, once these offspring had emerged, the foundress did not leave the nest, thus expressing the same behaviour as eusocial foundresses native to the Sussex site; and two of the three provisioning offspring were among the smallest produced by transplanted foundresses. Our limited data hint that sociality expressed by Inverness bees might be inefficient: despite the nest containing three provisioning B1 females (mean for native nests = 3.1 ± 0.33), productivity at this nest was lower than at any native nest that successfully produced B2 offspring (Fig. 3).

Phenotypic plasticity can be lost via genetic drift and subsequent genetic assimilation once environmental conditions become predictable (Masel et al. 2007; Pfennig et al. 2010), and when circumstances in which the alternative phenotype is expressed no longer arise (Sikkink et al. 2014; Smith et al. 2015; Cini et al. 2015). At Inverness, B1 females always enter hibernation, whereas at Sussex they may either become workers or enter hibernation (Davison and Field 2016). Plasticity could be lost in solitary populations where emerging offspring only ever receive cues associated with entering hibernation, such as reaching adulthood late in the season (Hirata and Higashi 2008; Field et al. 2010) or mating soon after reaching adulthood (Yanega 1989, 1997; but see Lucas and Field 2013). Therefore, at Inverness, loci regulating eusocial behaviour will not be exposed to selection because female offspring always enter hibernation. This could lead to genetic changes in the response threshold at which eusociality is expressed, and to its eventual loss from the population (Abouheif and Wray 2002; Suzuki and Nijhout 2006; Sikkink et al. 2014). Through this process, for example, the threshold at which bees from Inverness express eusociality might be higher than for native Sussex bees. In the UK, eusociality in sweat bees is restricted to the south (Soro et al. 2010; Falk 2015; Davison and Field 2016), and it is possible that *L. calceatum* from our Inverness population might exhibit greater plasticity if transplanted further south in Europe to sites where environmental cues for sociality are more extreme (Sikkink et al. 2014).

Local adaptation requires mechanisms that minimise gene flow between eusocial and solitary populations (Lenormand 2002). Without physical barriers to gene flow, one possibility could be differences in the timing of offspring production (Soucy and Danforth 2002; Quintero et al. 2014; Weis 2015). In sympatry, eusocial nests produce reproductive offspring in the second brood, later than the first brood reproductives produced in solitary nests. However, assortative mating may not occur because the first brood in eusocial nests often contains males, together with some females which may mate and enter hibernation without becoming workers (Plateaux-Quénu 1992; Davison and Field 2016; PJD, personal observation).

### Eusociality in *L. calceatum*

We confirmed that native *L. calceatum* exhibits eusociality at Sussex. Relatedness among B2 female brood within nests was high (*r* = 0.74), and foundresses surviving to the end of the season tended to monopolise reproduction. In common with other eusocial sweat bees, we also found no evidence that Sussex foundresses mated multiply (Crozier et al. 1987; Packer and Owen 1994; Mueller et al. 1994; Field et al. 2010, but see Soro et al. 2009), consistent with the hypothesis that monandry might help to facilitate the evolution of eusociality (Boomsma 2007; Hughes et al. 2008). Our result contrasts with a recent study of *H. scabiosae* Rossi, where relatedness among B2 females was considerably lower due to high rates of foundress turnover and frequent drifting between nests (Brand and Chapuisat 2016). We were unable to sample workers comprehensively; however, we did detect at least one likely case of drifting in which an alien B1 female produced a B2 female offspring. We documented no cases of natal workers laying B2 female brood, consistent with the idea that B1 females will reproduce in the presence of the foundress only if she is not their own mother (Paxton et al. 2002).

## Conclusion

The possibility that differences in social phenotype between eusocial and solitary populations of *L. calceatum* primarily reflect genetic differentiation will be of special interest for future studies investigating the genomics of sociality (e.g. Kocher et al. 2013). Few studies have examined the extent to which social polymorphism has promoted population differentiation (e.g. see Soucy and Danforth 2002; Zayed and Packer 2002; Soro et al. 2010), or considered whether polymorphism could facilitate ecological speciation (Rundle and Nosil 2005; Thibert-Plante and Hendry 2011). It would be interesting to transplant bees from a less northerly solitary population, where selection for plasticity may have persisted and bees may reveal a lower threshold for the expression of eusociality. Furthermore, because both eusocial and solitary nesting has been recorded at Sussex, and eusocial foundresses routinely pass through a solitary phase in spring prior to worker emergence, bees from Sussex may be more predisposed to exhibit plasticity if transplanted to Inverness. In general, the cornucopia of social variation exhibited by sweat bees demands that species are studied in detail throughout their geographic range, and in a variety of environmental contexts (Wcislo and Danforth 1997; Wcislo 1997).

## Electronic supplementary material

ESM 1 (CSV 8 kb)

ESM 2 (DOCX 102 kb)

ESM 3 (XLSX 19 kb)
