# Supplementary material for: Limited social plasticity in the socially polymorphic sweat bee Lasioglossum calceatum
Source: Behav Ecol Sociobiol. 2018 Mar 10;72(3):56. doi: 10.1007/s00265-018-2475-9 (PMC5845590; doi:10.1007/s00265-018-2475-9)
Supplement: Supplementary file 2 — (DOCX 102 kb) [file 265_2018_2475_MOESM2_ESM.docx]

Limited social plasticity in the socially polymorphic sweat bee *Lasioglossum calceatum*

Behavioural Ecology and Sociobiology

PJ Davison & J Field

University of Exeter

p.davison@exeter.ac.uk

| Locus | Multiplex | Annealing temperature (C) | Product length (bp) | Forward sequence | Reverse sequence |
| --- | --- | --- | --- | --- | --- |
| LM20 | 1 | 57 | 80-96 | TGTTCCCTCTTGCCTCC | AACCTTGAGACCGGTGC |
| Mala09 | 2 | 57 | 75-97 | GTTCCGCCAGCTTCTACC | GCAAACTAGTCCGTTAACTCGT |
| LMA24 | 2 | 57 | 143-154 | TCCTCGGACAAGGAGATACG | TTCGGGTACCGTTCAGTCTC |
| LMA36 | 1 | 57 | 163-195 | GGCCCTTCGACTTTGTTG | GAATCTCTGGGTGCTCTAACG |
| LMA40 | 2 | 57 | 145-189 | CGTTCGTTCGTTCGTTACTG | CAGAGTGCGTCGCTTGTTAG |
| LMA02 | 1 | 57 | 133-159 | CCGAGTTCATCAACATCCTC | TTGATTATCAGCGAGATGAGC |
| LMA53 | 1 | 57 | 217-235 | ACGCGGGATTACTTTCAATC | CCAATTATCGGGTGAAGGAG |
| LMA03 | 1 | 57 | 134-168 | AAAGCGTTGCGAGACACC | AGCATAATGGAAACCCAACG |
| LMA12 | 2 | 57 | 133-143 | CCAACCGAACACCAACTTTC | CTCCCGGGTTGTCATGTAAG |
| LMA29 | 2 | 57 | 191-230 | CTCGTCCCTCGTGTGACTC | GTATCGTGCGTGCGTGTC |

Table S1. Details of the microsatellite loci used in the study. See Parsons et al. 2017

|  |
| --- |

Table S2. Details of genetic diversity for the Sussex and Inverness populations separately and combined of *Lasioglossum calceatum* used in our study. N alleles, the number of unique alleles detected at each locus; H_o_, observed heterozygosity; H_e_, expected heterozygosity; HWE, Hardy Weinberg equilibrium reported as a p-value where non significance indicates no deviation from HWE.

| Locus > | LM20 | Mala09 | LMA24 | LMA36 | LMA40 | LMA02 | LMA53 | LMA03 | LMA12 | LMA29 | Mean |
| --- | --- | --- | --- | --- | --- | --- | --- | --- | --- | --- | --- |
|  | **Sussex** |  |  |  |  |  |  |  |  |  |  |
| N alleles | 6 | 8 | 3 | 8 | 14 | 10 | 8 | 9 | 5 | 7 | **7.8** |
| H_o_ | 0.475 | 0.475 | 0.633 | 0.550 | 0.728 | 0.630 | 0.451 | 0.545 | 0.593 | 0.692 | **0.577** |
| H_e_ | 0.563 | 0.591 | 0.593 | 0.565 | 0.890 | 0.642 | 0.654 | 0.747 | 0.683 | 0.668 | **0.660** |
| HWE (p) | 0.818 | 0.587 | 0.481 | 0.106 | 0.267 | 0.676 | 0.040 | 0.379 | 0.594 | 0.293 |  |
|  |  |  |  |  |  |  |  |  |  |  |  |
|  | **Inverness** |  |  |  |  |  |  |  |  |  |  |
| N alleles | 2 | 6 | 5 | 3 | 12 | 4 | 4 | 8 | 5 | 4 | **5.3** |
| H_o_ | 0.043 | 0.818 | 0.560 | 0.080 | 0.846 | 0.731 | 0.462 | 0.778 | 0.577 | 0.077 | **0.497** |
| H_e_ | 0.043 | 0.684 | 0.770 | 0.153 | 0.867 | 0.631 | 0.625 | 0.882 | 0.621 | 0.113 | **0.539** |
| HWE (p) | NA | 1.000 | 0.045 | 0.046 | 0.473 | 0.665 | 0.015 | 0.050 | 0.398 | 0.071 |  |
|  |  |  |  |  |  |  |  |  |  |  |  |
|  | **Combined** |  |  |  |  |  |  |  |  |  |  |
| N alleles | 6 | 10 | 6 | 9 | 19 | 10 | 9 | 11 | 6 | 9 | **9.5** |
| H_o_ | 0.379 | 0.516 | 0.615 | 0.438 | 0.757 | 0.654 | 0.454 | 0.570 | 0.589 | 0.538 | **0.551** |
| H_e_ | 0.475 | 0.629 | 0.688 | 0.688 | 0.906 | 0.719 | 0.768 | 0.758 | 0.703 | 0.753 | **0.709** |

Table S3. Details of the composition of genotyped native B2 brood

| Nest | # females | # males | Total genotyped brood | F present when excavated? | # in largest sib group | # not sibs (1) | # not sibs (2) |
| --- | --- | --- | --- | --- | --- | --- | --- |
| 4 | 11 | 2 | 13 | 0 | 10 | 1 | 2 (males) |
| 5 | 2 | 1 | 3 | 0 | 2 | 1 (male) | 0 |
| 9 | 0 | 6 | 6 | 0 | 5 (males) | 1 (male) | 0 |
| 17 | 3 | 0 | 3 | 1 | 3 | 0 | 0 |
| 20 | 4 | 0 | 4 | 0 | 3 | 1 | 0 |
| 24 | 3 | 2 | 5 | 0 | 4 | 1 (male) | 0 |
| 26 | 5 | 2 | 7 | 0 | 4 | 2 (males) | 1 |
| 32 | 5 | 3 | 8 | 1 | 8 | 0 | 0 |
| 36 | 5 | 3 | 8 | 0 | 7 | 1 (male) | 0 |
| 38 | 2 | 3 | 5 | 0 | 3 | 2 | 0 |
| 45 | 8 | 1 | 9 | 1 | 8 | 1 (male) | 0 |
| 49 | 4 | 1 | 5 | 0 | 4 | 1 | 0 |
| 58 | 1 | 1 | 2 | 1 | 1 | 1 (male) | 0 |
| 59 | 3 | 0 | 3 | 0 | 3 | 0 | 0 |
| 92 | 2 | 0 | 2 | 0 | 1 | 1 | 0 |

Fig. S1

Clustering of *Lasioglossum calceatum* foundresses from Inverness (red) and Sussex (green), and the offspring they produced. Individuals clustered strongly into two populations (K=2 determined by the Evanno method, delta K= 195.55), n=39 individuals. All individuals know to be from Inverness or Sussex were correctly assigned to each population. All putative offspring of transplanted Inverness foundresses excavated from beneath their nests were confirmed as belonging to the Inverness population. Three B1 females that initiated independent summer nests were confirmed as belonging to our Sussex population.

Sussex

Inverness

Probability of membership
